# Engagement of General Practice in an Australian Organised Bowel Cancer Screening Program: A Cross-Sectional Survey of Knowledge and Practice

**DOI:** 10.31557/APJCP.2020.21.7.2099

**Published:** 2020-07

**Authors:** Carol A Holden, Oliver Frank, Ming Li, Ramesh Manocha, Joanna Caruso, Deborah Turnbull, Richard L Reed, Caroline L Miller, David Roder, Ian Olver

**Affiliations:** 1 *South Australian Health and Medical Research Institute, Adelaide, Australia. *; 2 *Discipline of General Practice, Adelaide Medical School, University of Adelaide, Adelaide, and Oakden Medical Centre, Hillcrest, Australia. *; 3 *Cancer Epidemiology and Population Health, Cancer Research Institute, University of South Australia, Adelaide, Australia. *; 4 *Healthed Pty Ltd, New South Wales, Australia. *; 5 *School of Psychology, University of Adelaide, Adelaide, Australia. *; 6 *Discipline of General Practice, Flinders University, Adelaide, Australia. *; 7 *School of Public Health, University of Adelaide, Adelaide, Australia. *

**Keywords:** Colorectal cancer, mass screening, primary health care, quality improvement, quantitative

## Abstract

**Background:**

Understanding factors causing variation in family physicians/general practitioners (GPs) screening knowledge, understanding and support of organised population-based colorectal cancer (CRC) programs can direct interventions that maximise the influence of a CRC screening recommendation from a GP. This study aims to assess contextual factors that influence knowledge and quality improvement (QI) practice directed to CRC screening in Australian general practice.

**Methods:**

A convenience sample of anonymous general practice staff from all Australian states and territories completed a web-based survey. Multivariate analyses assessed the association between CRC screening knowledge and QI-CRC practice scores and patient, organisational and environmental-level contextual factors.

**Results:**

Of 1,013 survey starts, 918 respondents (90.6%) completed the survey. Respondents less likely to recommend FOBT screening had lower knowledge and QI practice scores directed to CRC screening. Controlling for individual and practice characteristics, respondents’ rating of the Australian National Bowel Cancer Screening Program (NBCSP) support for preventive care, attending external education, and sufficient practice resources to implement QI practice (generally) were the strongest factors associated with QI practice directed towards CRC screening. Knowledge scores were less amenable to the influence of contextual factors explored.

**Conclusion:**

More active engagement of family medicine/general practice to improve screening promotion could be achieved through better QI resourcing without changing the fundamental design of population-based CRC screening programs.

## Introduction

Population-based colorectal cancer (CRC) screening programs worldwide aim to reduce CRC mortality through early disease detection (Navarro et al., 2017). In Australia, the National Bowel Cancer Screening Program (NBCSP) was introduced incrementally from 2006, to be complete in 2020, when all Australians aged 50-74 years will receive immunochemical faecal occult blood test (FOBT) kits directly to their home every two years (National Bowel Cancer Screening Program, 2016; Australian Institute of Health and Welfare, 2019). However, 15-years on, the Australian participation rate remains suboptimal at 41% (Australian Institute of Health and Welfare, 2019), partly due to the phased implementation process (Flitcroft et al., 2010), and limited involvement of family physicians/general practitioners (GPs) (Grogan and Olver, 2014).

The NBCSP is administered nationally with family physicians/GPs indirectly expected to encourage participation, particularly for non-adherent patients, and follow-up and referral for diagnostic (colonoscopy) assessment (National Bowel Cancer Screening Program, 2016). A FOBT screening recommendation from a family physician/GP is the most influential factor in a patient’s participation in CRC screening (Cole et al., 2002; Zajac et al., 2010). Yet, variation in practitioner screening knowledge and attitude appears to influence their CRC screening practice (Perin et al., 2015; Dawson et al., 2017; Şahin and Aker, 2017), although Australian studies pre-date the NBCSP implementation (Youl et al., 2006).

Despite practitioner CRC screening surveys identifying individual and practice factors that moderate screening practice (National Cancer Institute (NCI); Damery et al., 2010; Perin et al., 2015; Şahin and Aker, 2017), and numerous systematic reviews (including meta-analyses) describing effective primary care interventions (Emery et al., 2014), there has been little research progress demonstrating sustained improvements in CRC screening participation in routine practice (Dodd et al., 2018). This may be related to limited recognition of the diverse contextual influencers that moderate the implementation of primary care interventions (Moore et al., 2015; Lau et al., 2016). Little is known about those contextual factors that may influence whether family physicians/GPs advise their eligible patients to be screened (Davis et al., 2018). We hypothesise that understanding those factors that influence practitioner CRC screening knowledge and practice will identify intervention points to increase FOBT screening recommendations. In the context of a broader quality improvement study, we surveyed Australian general practice to identify these influential factors. The findings will support a more coordinated effort to support the general practice workforce in improving CRC screening participation rates.

## Materials and Methods

We undertook a cross-sectional web-based survey, informed by a recent systematic scoping review (Holden et al., 2020), to explore individual, practice and contextual factors that might influence screening recommendations in Australian general practice. 

*Participants*

An opportunistic, convenience sample of general practice staff was surveyed using a sampling frame of practitioner email addresses, registered with an education provider (Healthed Pty Ltd), who consented to receive email communication from the provider. A chance to win one of five $AUS100 gift vouchers was offered to those that completed the survey (Vangeest et al., 2007).

*Data collection*

Study data (predominately categorical responses and 5-point Likert scales) were collected and managed using REDCap (Research Electronic Data Capture), a secure, web-based application (Harris et al., 2009). A valid response was needed for survey completion with no constraints imposed on the response range for three knowledge-based and two open-response questions. The survey was open from 24 September to 10 October 2018, with one reminder sent to non-respondents after 10 days. The 50-item survey took on average less than five minutes to complete.

*Survey instrument*

Survey questions (Supplementary Data 1) were sourced primarily from the US National Cancer Institute Survey of Colorectal Cancer Screening Practices, and modified by Damery and colleagues (UK) (Damery et al., 2010), and as relevant for the Australian context (Britt et al., 2015). Questions included knowledge of Australian CRC screening guidelines (Cancer Council Australia Colorectal Cancer Guidelines Working Party, 2017), quality improvement (QI) practices, both general and specifically directed to colorectal cancer (QI-CRC) (The Royal Australian College of General Practitioners (RACGP), 2016), screening participation and individual socio-demographic (e.g. age) and practice characteristics (e.g. practice size). Geographical setting (Australian Statistical Geography Standard (ASGS) remoteness structure) (Australian Bureau of Statistics (ABS), 2018) and relative socio-economic disadvantage (Socio-Economic Indexes for Areas, SEIFA) (Australian Bureau of Statistics (ABS), 2016) were generated from practice postcode. 

Eight GPs from a large family/general practice initially tested the survey for design, content and completion time. A random sample of 18 GPs from metropolitan practices then pilot tested the survey for comprehension and response logic before final dissemination.

*Study outcome*

CRC screening ‘knowledge’, and ‘QI practice’ scores were calculated for each respondent based on the responses to three specific questions for each study outcome (Supplementary Data 2). Knowledge scores were dichotomised between those with a maximum possible knowledge score of nine and those scoring less (gaps in knowledge). QI-CRC screening practice score was dichotomised between those who were active in QI-CRC activity (score = 7 – 9) and those scoring less (low-moderate QI-CRC activity, < 7).

*Study factors*

FOBT screening recommendation and potential drivers (contextual factors) that might be associated with knowledge and QI practice directed to CRC screening recommendation were studied. Responses to ‘how often do you recommend FOBT for your asymptomatic, average-risk patients’ were dichotomised between ‘always’ and ‘not always’ (responses other than ‘always’).

A three-level (patient, organisation and environmental) complexity framework of contextual factors, modified from Lau et al. (Lau et al., 2016), was applied with selected factors identified from the literature (Holden et al., 2020):

i) patient-level factors (practice demographic profile (Davis et al., 2018)): respondents understanding of their patient profile for the targeted age-group (aged 50 years or older) and under-screened population subgroups (e.g. Aboriginal and/or Torres Strait Islander clients; low socio-economic status, SES).

ii) organisation/system-level factors (existing QI resources and activity (Arroyave et al., 2011)): for each respondent, a total QI activity score (range, three to nine) was calculated from their self-reported use of data extraction tools, measurement and discussion of practice performance data (Supplementary Data 2). QI scores were categorised into three groups reflective of their general QI-activity level: low QI activity (score = 3); medium QI activity (score = 4 – 6); active QI (score = 7 – 9).

iii) environmental-level factors (infrastructure (i.e. NBCSP) (Tong et al., 2004), financial incentives (Mauro et al., 2019) and external education activities): respondents rated the extent that the NBCSP improves care (Unsatisfactory, <40; Neutral, 40-60; Significantly improves care, >60), the influence of a financial incentive (Australian Government Practice Incentives Program, PIP) on CRC screening participation and attendance at quality care/prevention education activities in the past two years.

*Statistical analyses*

The mean (and standard deviation) knowledge and QI-CRC practice score was presented and compared by respondent sociodemographic and practice characteristics and study factors measured using parametric or non-parametric tests as appropriate. 

The association (OR) between knowledge and QI-CRC practice and the study factors measured was assessed using logistic regression analysis. The adjusted association was from models including respondents’ sociodemographic and practice characteristics. In addition, a step-wise logistic regression analysis was conducted by including all measured study factors and covariates to identify factors significantly associated with knowledge and QI-CRC practice. 

All analyses were conducted using STATA 14 (STATACorp, College Station, Texas, USA). Results were considered statistically significant at p<0.05.

*Ethics approval*

This survey was part of a larger study approved by the University of South Australia Human Research Ethics Committee (Application ID: 201061). Participants choosing to complete the online survey were deemed as providing consent, as approved by the ethics committee. 

## Results

Of the 1,013 recipients who started the survey, 918 surveys were available for analysis. According to the American Association for Public Opinion Research (AAPOR) (American Association for Public Opinion Research, 2016), this yields a Cooperation Rate 4 (COOP4), which includes partial responses, of 90.6%. There were no significant differences between the ‘Incomplete’ and ‘Complete’ survey respondents in general practice characteristics (Supplementary Data 3).

Respondent, individual and practice characteristics are presented in Table 1. Most respondents were female, aged over 55 years, completed their medical training in Australia and had personal experience of CRC screening: for those who were screened, 40% (n=218) were screened via FOBT only, 30% (n=166) via FOBT and colonoscopy, and 30% (n=164) via colonoscopy only. Respondents worked in practices from all states and territories, with most working in practices in major cities and a gradient in the socio-economic practice setting. Most respondents knew their practice demographic relating to age and socio-economic status, however fewer knew their practice proportion of under-screened populations (e.g. Aboriginal and Torres Strait Islander clients, non-English speaking patients and new migrants/refugees). 

Respondent characteristics were different from the Australian general practice workforce (Commonwealth Department of Health) (Supplementary Data 4). When compared to other Australian studies where a small proportion of general practice respondents were drawn from a larger workforce sampling frame (e.g. BEACH data (Britt et al., 2015)), respondents were more likely to be female (*χ*^2^ 247.85, p<0.001) and from different states (*χ*^2^ 19.24, p=0.01) (Supplementary Data 4).

*Colorectal cancer (CRC) screening knowledge and QI-CRC practice*

Mean knowledge scores about CRC screening were higher for females, younger respondents (both in years of practice and in age), those who had not previously been screened and respondents from group practices (Table 1). QI practices relating to CRC screening (QI-CRC) were not different by practice characteristics, however male respondents, Australian-trained respondents and those with fewer years of practice had significantly better QI-CRC practice scores than their respective counterparts (Table 1).

*FOBT screening recommendation and knowledge and QI-CRC practice*

Half of respondents ‘always’ recommended FOBT to their eligible patients (n=424, 50%). About a third of respondents always/very often recommended colonoscopy (n=308, 36%) despite no Australian guidelines recommending these tests as screening procedures for asymptomatic, average-risk patients. 

Those respondents who ‘always’ recommended FOBT to their asymptomatic, average-risk patients had higher mean scores for knowledge (8.2 (SD**±**0.8) vs 8.0 (SD **±**1.0), p<0.013) and QI-CRC practice (6.3 (SD**±**2.0) vs 5.6 (SD**±**1.9), p<0.001), than those who did not ‘always’ recommend FOBT screening to their eligible patients. 

*Contextual factors and their association with CRC screening knowledge and QI-CRC practice*

Lower knowledge scores were observed in practices not servicing the target population (>50 years) (Table 2). Servicing under-screened population groups did not appear to influence CRC screening knowledge scores (Table 2 and Table 3). Similarly, none of the organisation-level or environmental-level factors explored influenced knowledge scores (Table 2 and Table 3), except that a financial incentive would not make a difference to CRC screening practice for those respondents with higher knowledge scores (Table 3).

In contrast, while patient-level factors did not appear to influence QI-CRC practice scores, organisational and environmental-level factors had varying influence on active QI-CRC practice. Those respondents who reported having insufficient resources to implement QI programs, reported lower QI-CRC practice scores (Table 2) after adjusting for individual and practice characteristics (Table 3). Similarly, environmental-level factors had a positive influence on QI-CRC practice: respondents who positively rated the NBCSP as effectively supporting preventive care, and those respondents who recently completed external education, reported significantly higher QI-CRC practice scores (Table 2) and these activities increased the likelihood of active QI-CRC practice (Table 3). A small proportion of respondents with lower QI-CRC practice scores indicated that a financial incentive would make a difference to their CRC screening practice (Table 2) but this association did not persist when controlling for individual and practice characteristics (Table 3).

In the final full step-wise logistic regression model, factors significantly associated with higher CRC screening knowledge and QI-CRC practice score included younger respondents (<55 years) who were 130% more likely (95% CI 1.7-3.1), while those who indicated that a financial incentive would not influence their screening practice were 30% less likely (OR 0.7, 95% CI 0.5-0.9) to achieve full knowledge scores. Being active in QI-CRC was more common among male respondents (OR 1.7, 95% CI 1.2-2.4), those practicing for less than 20 years (OR 1.5, 95% CI 1.1-2.0) and respondents who positively rated the NBCSP (OR 1.5, 95% CI 1.1-2.1), had sufficient resources (OR 2.1, 95% CI 1.4-3.2), were actively implementing QI practice (OR 2.0, 95% CI 1.4-3.2) and who had within the past two years attended external education activities (OR 1.7, 95% CI 1.1-2.8).

**Table 1:** Knowledge and Quality Improvement Practice Scores Directed to Bowel Cancer Screening by Respondent Socio-Demographic and Practice Characteristics

Socio-demographic characteristic	Number (%)	Knowledge Mean Score (SD)		QI-CRC Practice Mean Score (SD)	
Gender			p=0.003		p=0.009
Male	171 (20%)	7.9 (0.9)		6.3 (2.0)	
Female	647 (79%)	8.1 (0.9)		5.8 (2.0)	
Invalid/prefer not to say	4 (1%)	7.9 (0.9)		4.8 (2.9)	
Age			p<0.001		p=0.704
<35 years	74 (9%)	8.4 (1.0)		6.1 (2.0)	
35-44 years	147 (18%)	8.4 (0.7)		5.9 (1.9)	
45-54 years	192 (24%)	7.9 (0.9)		5.8 (2.1)	
55+ years	403 (49%)	7.9 (0.9)		6.0 (2.0)	
Country of Graduation			p=0.878		p<0.001
Australia	530 (70%)	8.1 (0.9)		5.7 (1.9)	
Overseas	222 (30%)	8.1 (0.8)		6.5 (2.0)	
Years of practice (years)			p<0.001		p=0.057
< 5	153 (19%)	8.4 (0.9)		6.2 (2.0)	
6-10	130 (16%)	8.2 (0.8)		6.0 (1.9)	
11-19	109 (13%)	8.2 (0.8)		6.1 (2.1)	
20+	428 (52%)	7.9 (0.9)		5.7 (2.0)	
Personally screened			p<0.001		p=0.618
Screened	549 (65%)	8.0 (0.9)		5.9 (2.0)	
Not screened	283 (33%)	8.3 (0.8)		5.9 (2.0)	
Prefer not to say	18 (2%)	7.9 (0.7)		6.4 (1.9)	
Practice characteristic	Number (%)	Knowledge Mean Score (SD)		QI-CRC Practice Mean Score (SD)	
Nos. GPs working at practice			p= 0.002		p= 0.330
Sole provider	45 (5%)	7.6 (1.2)		6.2 (2.1)	
2-3	165 (20%)	8.0 (0.9)		6.1 (2.1)	
4-9	444 (53%)	8.2 (0.8)		5.9 (2.0)	
10+	190 (23%)	8.2 (0.8)		5.8 (1.9)	
Nos. nursing staff (FTE)			p= 0.460		p= 0.414
Nil	79 (9%)	7.9 (1.0)		5.6 (2.1)	
<1.0	287 (34%)	8.2 (0.8)		6.0 (1.9)	
1.0-3.0	350 (41%)	8.1 (0.8)		5.9 (2.0)	
3.1-6.0	110 (13%)	8.0 (0.9)		6.2 (2.0)	
6.1+	22 (3%)	8.2 (0.9)		5.9 (2.1)	
Practice ownership model			p= 0.011		p= 0.427
GP-owned	591 (70%)	8.1 (0.8)		5.9 (1.9)	
Corporate practice	138 (16%)	8.1 (0.9)		5.9 (1.9)	
Community-controlled	53 (6%)	8.1 (1.0)		6.0 (1.9)	
Other	67 (8%)	7.7 (1.1)		5.5 (2.1)	
Relative Socio-economic disadvantage index quintile			p= 0.720		p= 0.214
1st (most disadvantage)	98 (12%)	8.0 (0.9)		6.0 (2.1)	
2-5	147 (18%)	8.0 (0.9)		6.0 (2.1)	
6-10	152 (18%)	8.1 (0.8)		6.1 (1.9)	
11-19	148 (18%)	8.1 (0.9)		5.7 (2.0)	
5th (least disadvantage)	294 (35%)	8.1 (0.9)		5.8 (1.9)	
ASGS - Remoteness Assessment (practice setting)			p= 0.525		p= 0.232
Major cities	617 (74%)	8.1 (0.9)		5.9 (2.0)	
Inner regional	146 (17%)	8.1 (0.8)		6.2 (2.0)	
Outer regional or remote	76 (9%)	8.2 (0.9)		5.9 (2.1)	

Data presented as mean (SD); *p*- value from t-test or one-way ANOVA

**Table 2:** Knowledge and Quality Improvement Practice Scores Directed to Bowel Cancer Screening and Association with Selected Contextual Factors

Contextual factors	Number	Knowledge Mean Score (SD)		QI-CRC Practice Mean Score (SD)	
*Patient-level factors *					
Demographic: 50+ yr olds			p< 0.001		p= 0.885
None	7 (1%)	6.3 (1.3)		6.0 (2.3)	
Low-Moderate	820 (97%)	8.1 (0.9)		5.9 (2.0)	
I don’t know	20 (2%)	8.1 (0.8)		6.2 (1.8)	
Demographic: Aboriginal and Torres Strait Islander			p= 0.463		p= 0.060
None	413 (49%)	8.1 (0.9)		5.8 (2.0)	
Low-Moderate	236 (28%)	8.1 (0.8)		6.1 (2.1)	
I don’t know	192 (23%)	8.0 (0.9)		6.0 (2.0)	
Demographic: Low SES			p= 0.031		p= 0.799
None	89 (11%)	7.9 (1.1)		6.1 (1.9)	
Low-Moderate	706 (84%)	8.1 (0.8)		5.9 (2.0)	
I don’t know	50 (6%)	8.0 (0.8)		5.8 (1.9)	
*Organisational-level factors*					
Sufficient QI resources			p= 0.903		p< 0.001
Insufficient	91 (11%)	8.1 (0.8)		5.0 (1.9)	
Neutral	177 (21%)	8.1 (0.9)		5.3 (1.8)	
Sufficient	585 (69%)	8.1 (0.9)		6.3 (2.0)	
QI activity level			p= 0.289		p< 0.001
Low level QI	302 (35%)	8.2 (0.9)		5.1 (1.7)	
Medium QI	330 (39%)	8.0 (0.9)		5.9 (1.9)	
Active QI	221 (59%)	8.0 (0.9)		7.1 (1.8)	
*Environmental-level factors*					
NBCSP Rating Score			p= 0.387		p< 0.001
Unsatisfactory	90 (11%)	8.2 (0.8)		4.4 (1.8)	
Meets expectations	257 (31%)	8.0 (1.0)		5.2 (2.1)	
Significantly improves care	491 (59%)	8.1 (0.9)		5.2 (2.1)	
PIP payment			p= 0.130		p= 0.058
Would make no difference	593 (71%)	8.1 (0.8)		6.0 (2.0)	
Neutral	162 (20%)	8.1 (1.0)		5.8 (1.8)	
Would make a difference	75 (9%)	7.9 (1.0)		5.5 (2.1)	
External education			p= 0.67		p= 0.01
Yes	680 (80%)	8.1 (0.9)		6.0 (2.0)	
No	99 (12%)	8.1 (0.8)		5.4 (1.8)	
I don’t remember	71 (8%)	8.0 (1.1)		5.8 (2.1)	

Data presented as mean (SD); *P*- value from t-test or one-way ANOVA

**Table 3:** Results of Logistic Regression Models Estimating the Association between Selected Contextual Factors and Full Knowledge Score* and Active QI Practice** for Bowel Cancer Screening in Australian General Practice

	Full knowledge score*		Active QI practice**	
	Number (%)	Adjusted OR (95% CI)	Number (%)	Adjusted OR (95% CI)
Demographic: 50+ yr olds				
None	0	NA	3 (0.8%)	0.8 (0.1 - 11.0)
Low-Moderate	319 (97.9%)	Reference	349 (96.4%)	Reference
I don’t know	7 (2.2%)	0.5 (0.7 - 1.8)	10 (2.7%)	1.6 (0.5 - 5.2)
Demographic: Aboriginal and Torres Strait Islander				
None	156 (48.5%)	1.0 (0.7 - 1.6)	163 (45.7%)	0.8 (0.5 - 1.2)
Low-Moderate	97 (30.1%)	Reference	108 (30.3%)	Reference
I don’t know	69 (21.4%)	1.0 (0.6 - 1.6)	86 (24.1%)	1.1 (0.7 - 1.7)
Demographic: Low SES				
None	29 (9%)	0.7 (0.4 - 1.3)	43 (11.9%)	1.5 (0.9 - 2.5)
Low-Moderate	281 (87.0%)	Reference	297 (82.5%)	Reference
I don’t know	13 (4.0%)	0.4 (0.2 - 1.0)	20 (5.6%)	0.9 (0.4 – 1.8)
Sufficient QI resources				
Insufficient	34 (10.4%)	1.1 (0.6 - 2.0)	20 (5.5%)	0.9 (0.4 - 1.7)
Neutral	69 (21.0%)	Reference	47 (13.0%)	Reference
Sufficient	225 (68.6%)	1.2 (0.8 - 1.8)	296 (81.5%)	3.0 (2.0 - 4.6)
QI practice				
Low level QI	122 (37.2%)	0.7 (0.5 - 1.1)	73 (20.1%)	0.1 (0.1 - 0.2)
Medium QI	118 (36.0%)	0.8 (0.5 - 1.2)	148 (40.8%)	0.4 (0.3 - 0.6)
Active QI	88 (26.8%)	Reference	142 (39.1%)	Reference
NBCSP Rating Score				
Unsatisfactory	35 (10.9%)	1.1 (0.6 - 2.0)	25 (6.9%)	0.7 (0.4 - 1.2)
Meets expectations	94 (29.3%)	Reference	98 (27.0%)	Reference
Significantly improves care	192 (59.8%)	1.2 (0.8 - 1.7)	240 (66.1%)	1.6 (1.1 - 2.2)
PIP payment				
Would make no difference	221 (69.5%)	0.6 (0.4 - 0.9)	269 (74.1%)	1.2 (0.8 - 1.8)
Neutral	73 (23.0%)	Reference	66 (18.2%)	Reference
Would make a difference	24 (7.6%)	0.5 (0.3 - 1.1)	28 (7.7%)	0.9 (0.5 -1.8)
External education				
Yes	270 (82.3%)	Reference	302 (83.2%)	Reference
No	31 (9.5%)	0.7 (0.4 - 1.1)	33 (9.1%)	0.6 (0.3 - 0.9)
I don’t remember	27 (8.2%)	1.2 (0.7 - 2.1)	28 (7.7%)	0.8 (0.5 - 1.4)

*, Full knowledge score is those respondents who scored 9 (Supplementary Data 2); **, Active quality improvement directed to bowel cancer screening is those respondents who scored 7 to 9 (Supplementary Data 2)

## Discussion

Our study identified contextual factors that influenced active QI-CRC practice, which indirectly influenced frequency of FOBT recommendations, but did not appear to influence CRC screening knowledge. Most notably, rating the NBCSP as supporting preventive care, attending external education activities and having sufficient resources to implement QI practice (general) were the contextual factors most strongly associated with QI-CRC practice. 

We believe that this is the first quantitative evaluation of CRC screening practice in Australian general practice, since the NBCSP was implemented in 2006. While studies suggest that GP knowledge of screening guidelines may influence population-based screening (Dawson et al., 2017), our findings highlight that FOBT screening recommendations are more varied and complex than the underlying CRC screening knowledge would suggest. Indeed, ‘QI practice’ may have a greater influence on the frequency of FOBT recommendations than ‘knowledge’, which does not appear to be as strongly influenced by the contextual factors explored.

Practitioner perception, understanding and support of an organised population-based screening program may influence its success (Aubin-Auger et al., 2011; Dawson et al., 2017; Dimova et al., 2018), even when the program operates independently of general practice (Grogan and Olver, 2014). Few improvements in CRC screening participation in practice have been reported (Dodd et al., 2018), despite evidence describing effective interventions to enhance screening participation in primary care (Emery et al., 2014). This study begins to identify moderating contextual factors, which little is known (Davis et al., 2018), that potentially account for the variation in practitioner screening knowledge and QI-CRC practice that appear to indirectly influence FOBT screening recommendations in general practice (Perin et al., 2015; Dawson et al., 2017; Şahin and Aker, 2017). The multi-level (patient, organisation and environmental) complexity framework applied is a unique approach to exploring potential contextual predictors of FOBT recommendation, via knowledge and practice improvements. Our study suggests that environmental-level strategies beyond the clinical practice setting (e.g. federal support of general practice, NBCSP enhancements) may enhance the family physician/GP role in encouraging patients to participate in CRC screening. Such a finding also overcomes the potential implementation barriers frequently encountered in primary care (Moore et al., 2015; Lau et al., 2016).

Better support for QI practice opportunities particularly for those under-resourced practices, such as the NBCSP engaging directly with general practice for example, through the National Cancer Screening Register (Australian Government Department of Health) and Quality Improvement Incentive - Practice Incentives Program (Australian Government Department of Human Services) might facilitate the integration of existing clinical practice with NBCSP activity. QI practice is a fundamental element of quality/preventive care and tends to be more effective in achieving change in routine clinical practice (Grol and Grimshaw, 2003; Holden et al., 2020) and would potentially enhance the practitioner role in the NBCSP without altering the program design. 

GPs were not involved in the initial design of the Australian NBCSP program (Grogan and Olver, 2014), which may be reflected in some ‘unsatisfactory’ NBSCP rating scores in our study (Tong et al., 2004). While efforts are now being made in Australia to better engage the primary health care community (National Bowel Cancer Screening Program), an integrated approach is needed to enhance the fundamental general practice role in preventive and quality care aligned to population-based CRC screening. While individual and practice attributes might indirectly influence the effectiveness of primary care CRC screening interventions, focusing support on high-level systematic QI practice may indirectly improve FOBT screening participation, a hypothesis that warrants further investigation. 

Study limitations include the opportunistic email recruitment of a difficult group to recruit (Vaisson G et al., 2018), resulting in a diverse but not necessarily representative sample of practitioners despite measures to maximise the response in the invitation and survey design (Petrovčič et al., 2016). However, respondents are comparable to responders to other similar Australian surveys (Britt et al., 2015) and the sample population is relatively heterogeneous, with most variables showing a diverse response spread allowing comparisons within the cohort. While we found small absolute differences in knowledge scores, the dataset is sufficiently robust to identify step-wise trends and significant associations with contextual factors explored in this study. Such a large and variable sample from every state and territory, has permitted us to canvass a broad range of practice and perspectives being indicative of CRC screening in routine practice. 

The study also demonstrates the transferability of survey questions used in other regions (US and UK (Damery et al., 2010)) and modified for the Australian context and its suitability for repeated use. Although the survey methodology does not allow in-depth analysis or association direction (Moore et al., 2015), including the evaluation of targeted general practice interventions and ongoing approval rating of organised CRC screening programs may provide organised screening programs with an indirect indicator of FOBT screening recommendations in general practice. 

Our quantitative study has addressed the gap in understanding of contextual factors that indirectly influence CRC screening recommendations in general practice. The findings are most relevant to population-based CRC screening programs where general practice has an influential role in supporting program delivery and recommending CRC screening participation, particularly for non-adherent or under-screened patients.
